# Evaluating the Possibility of Transfusion Medicine, Through Crossmatching in Juvenile Galapagos Sea Lions (*Zalophus wollebaeki*)

**DOI:** 10.3389/fvets.2022.830272

**Published:** 2022-04-20

**Authors:** Taylor M. Gregory, Maryanna Parker, Diane Deresienski, Daniela Alarcón-Ruales, Juan Pablo Muñoz-Pérez, Jorge Torres, Gabriela I. Gavilanes, Gregory A. Lewbart, Diego Páez-Rosas

**Affiliations:** ^1^Department of Clinical Sciences, North Carolina State University, Raleigh, NC, United States; ^2^USFQ & UNC-Chapel Hill Galápagos Science Center (GSC), Av. Alsacio Northia, Isla San Cristobal, Galápagos, Ecuador; ^3^Universidad San Francisco de Quito (USFQ), Colegio de Ciencias Biológicas y Ambientales, Galápagos, Ecuador; ^4^USFQ & UNC-Chapel Hill Galápagos Science Center (GSC), Galápagos, Ecuador; ^5^Dirección Parque Nacional Galápagos, Oficina Técnica San Cristobal, Galápagos, Ecuador

**Keywords:** sea lion, Galapagos archipelago, blood, crossmatch, transfusion

## Abstract

The Galapagos sea lion (*Zalophus wollebaeki*) is an endemic pinniped to the Galapagos archipelago, and like most wild mammals, is at risk for anemia due to trauma, infectious disease, and poor nutrition. This study evaluated the health status of 26 juvenile Galapagos sea lions on the island of San Cristobal prior to evaluating 100 crossmatch combinations. On evaluation, all but one sea lion had no major systemic abnormalities. Of the 100 crossmatches performed, 23% had minor reactions. The most significant reaction was weak macroscopic agglutination found in 4% of samples. The small percentage of agglutination reactions suggests a small proportion of naturally occurring alloantibodies in this species and may be consistent with a low risk of acute immune-mediated hemolytic transfusion reaction.

## Introduction

Galapagos sea lions (*Zalophus wollebaeki*) are one of two endemic pinnipeds in the Galapagos archipelago. Due to a concerning decline in the population over the past 30 years, this species has been listed as endangered on the IUCN red list (1). The main factor in this population decline is the variable availability of resources during oceanographic warming events such as the El Niño–Southern Oscillation (2, 3). However, the risk of exposure to diseases related to introduced animals (e.g., dogs and cats) and injuries associated with anthropogenic activities increases as the human population and tourism industry grow (4, 5).

Trauma and chronic diseases can both cause life threatening anemias in mammals (6, 7). The effects of diseases-related secondary anemia in Galapagos sea lions are unknown; however, pinnipeds have been diagnosed with various parasitism diseases (e.g., trematodes and acanthocephalans) causing secondary anemia (8, 9). Additionally, anthropogenic impacts (e.g., boat impacts) are one of the two main threats to marine mammals and can cause significant blood loss (10). Given the increasing likelihood of anemia in Galapagos sea lions, more methods are needed to manage this potential disease in this species.

In the timeline of veterinary medicine, transfusion medicine is relatively new. Thus, it has only been extensively studied in domestic animals and comparatively few studies have looked into transfusion medicine and blood types in wildlife and zoo species (11–13). With the increased interest in zoo and wildlife species transfusion medicine (14, 15), evaluating the possibility of blood transfusions will further knowledge of wildlife medicine. Therefore, the objective of this study was to perform the first blood crossmatching of the endangered Galapagos sea lion (*Zalophus wolleabeki*), to provide information that facilitate transfusion medicine in this species and other pinnipeds.

## Materials and Methods

### Animal Use Statement

This study was conducted and supported by the Galapagos National Park Directorate (GNPD) and the Universidad San Francisco de Quito (USFQ) under research permit PC-31-21. The fieldwork and sample collection were carried out following the protocols of ethics and animal handling approved by the GNPD and USFQ. All sea lions sampled during this study were handled based on current standards of care. The animals of this study were not used for any other hematological analysis during the period in which the study was completed.

### Animal Care

All animals utilized in this study were wild and not under human care. They were monitored by a veterinarian and a Galapagos National Park rangers during this study.

### Animal Selection and Capture

Non-nursing juvenile animals from the El Malecón rookery in San Cristobal Island were selected (Playa Mann Day 1, Playa Los Marinos Day 2), which is the largest and most representative rookery in the archipelago (3). Each sea lion was captured by placing a capture net in front of the sea lion and encouraging them into the net.

### Morphometric Data Collection

At time of capture, the sea lion was weighed using an electronic scale. The weight of the net was then subtracted from the total weight to determine the real weight of each sea lion. Subsequently, each animal was removed from the net and manually held by three experts in handling this species. This procedure is quick (<10 min) and consists of covering the head with a wet towel while holding the neck, back and fins to prevent the animal from moving and remain in a passive state. A physical examination was performed by a veterinarian and routine morphometric measurements were taken.

### Blood Sampling and Processing

Depending on the size and weight of the animal, either a 20 G or 18 G 1.5-inch needle was utilized for venipuncture. The needle was heparinized with lithium heparin. The caudal gluteal vein was used for all venipuncture sites. The site was cleaned with alcohol prior to collection. Up to 3 milliliters of blood was collected from each animal and immediately placed in a heparinized tube. After each collection, the samples were allowed to cool and placed on a towel in a cooler with blue ice until processing.

Processing occurred on the same day as sampling. The blood samples were used for measuring various hematological parameters following the protocol used in this species by Páez-Rosas et al. (16). Blood lactate was determined using a portable Lactate Plus^TM^ analyzer (Nova Biomedical, Waltham, Massachusetts, USA). The i-STAT Clinical Analyzer (Heska Corporation, Fort Collins, Colorado, USA) is a handheld device that measures selected blood gas, biochemical, and hematology parameters using ~0.095 ml of non-coagulated whole blood. The following parameters were obtained on the Chem8 cartridge: base excess in the extracellular fluid compartment (BEECF), bicarbonate (${\text{HCO}}_{3}^{-}$), glucose, hematocrit (HCT), hemoglobin (Hgb), ionized calcium (iCa), partial pressure of carbon dioxide (pCO_2_), total carbon dioxide (TCO_2_), partial pressure of oxygen (pO_2_), pH, potassium (K), and sodium (Na). About 0.05 ml was used for centrifugation with a portable microcentrifuge (Eppendorf North America, Inc., centrifuge model 5,424, 5 min. at 14,000 G) to determine packed cell volume (PCV) and total solids (TS). Two drops of plasma were placed on a refractometer (Ade Advanced Optics, Oregon City, Oregon, USA) and the total solids values recorded.

In preparation for crossmatching, all samples were allowed to warm to room temperature. The heparinized tubes were placed in a centrifuge (Gemmy Industrial Corporation, Taipei, Taiwain) for 10 min at 3,500 rpm. The plasma was then pulled and placed into an individual glass tube 12 × 75 mm labeled for each animal. One hundred microliters of red blood cells were drawn from the centrifuged tubes, and care was taken to avoid aspirations of the buffy coat. These red blood cells were placed in an individual tube labeled for each sea lion. The red blood cells were then washed by filling the tube ¾ of the way with sterile saline and then mixing with gentle inversion. After the cells were mixed, they were centrifuged at 3,500 rpm for 60 s. The supernatant was decanted and then the previous steps were repeated twice. After the third wash, a 3% solution was made by adding 3.2 ml of saline to the red blood cell pellet.

Each sea lion was then crossmatched to itself by placing two 75 microliter drops of plasma and one 75 microliter drop of its red blood cell solution into a new labeled glass tube. The tube was mixed and incubated at 37 degrees C for 30 min. After incubating, each tube was centrifuged for 20 s at 3,500 rpm. The contents were then examined macroscopically and microscopically for signs of clotting. Table 1 defines the evaluation score for each crossmatch. The crossmatching method took ~45 to 60 min to complete.

**Table 1:** Description of microscopic and macroscopic transfusion reactions.

**Reaction grade**	**Description**
Negative reaction	Intact red blood cells evenly dispersed throughout fluid solution. No evidence of cell lysis or clotting.
Hemolysis	Lysed red blood cells in serosanguinous solution. No evidence of intact red blood cells or clotting.
m+_	A microscopic agglutination reaction. Agglutinated aggregates of red blood cells not visible to the naked eye, but only observable under 10 × magnification.
W+	A weak agglutination reaction. Multiple pinpoint agglutinated aggregations of red blood cells among single, non-agglutinated red blood cells. No evidence of cell lysis.
1+	Multiple small agglutinated aggregations of red blood cells without single, non-agglutinated red blood cells. No evidence of cell lysis.
2+	Multiple medium sized agglutinated aggregations of red blood cells without single, non-clotted red blood cells. No evidence of cell lysis.
3+	A single large agglutinated aggregation of all red blood cells without single, non-clotted red blood cells. No evidence of cell lysis.

On day one of sampling, due to the low number crossmatching possibilities, each sea lion was paired with every other sea lion sampled during the day. Due to the increased number of crossmatching possibilities on the second day, simple random sampling was utilized to select the crossmatching pairs. Each pair had two crossmatches run (major and minor) with one sea lion assigned as the recipient and one as the donor. For the major crossmatch, two 75 microliter drops of recipient plasma and one 75 microliter drop of the donor's red blood cell solution was placed into a new labeled glass tube. For the minor crossmatch, two 75 microliter drops of donor plasma and one 75 microliter drop of the recipient's red blood cell solution was placed into a new labeled glass tube. Both tubes were mixed and incubated at 37 degrees C for 30 min. After incubating for 30 min, each tube was centrifuged for 20 s at 3,500 rpm. The contents were then examined macroscopically *via* a handheld magnifying glass (IAMGLOBAL, Calabasas, California, USA) and microscopically underneath a microscope (AmScope, Irvine, California, USA) at 10 × magnification for agglutination. All macroscopic and microscopic examination was performed by a single researcher to limit subjective bias within the study.

## Results

A total of 26 juvenile sea lions were captured over a two-day period. Twelve animals were captured at Playa Mann while 14 animals were captured at Playa Los Marinos. Animal restraint time was 10.6 min on average. There were 14 male and 12 females with an age range of 2 to 5 years. The most common abnormality on physical examination was presence of ocular trematodes (10/26; 38%). Other abnormalities on physical examination included minor traumatic injuries (lacerations and scars) (3/26; 11.5%), a ruptured globe (1/26; 3.8%), and thin body condition (1/26; 3.8%). Routine bloodwork was assayed to determine the health of 21 animals (8 animals from Playa Mann and 13 animals from Playa Los Marinos) and is summarized in Table 2.

**Table 2:** Summary of hematological parameters evaluated in 21 juvenile Galapagos sea lions (*Zalophus wollebaeki*).

**Parameter**	**Mean**	**SD**	**Minimum**	**Maximum**
Na (mmol/L)	147	3.1	136	151
K (mmol/L)	3.9	0.5	2.9	5
Cl (mmol/L)	112.9	3.3	107	120
TCO_2_ (mmHg)	23.7	5.2	15	31
Anion gap	15.2	5.0	0	22
iCa (mmol/L)	1.1	0.2	0.6	1.28
BUN [mg/dl (mmol/L)]	36.0 (2.00)	23.7 (1.32)	9 (0.50)	102 (5.66)
Crea [mg/dl (mmol/L)]	0.6 (0.03)	0.2 (0.01)	0.2 (0.01)	1 (0.06)
HCT (%)	53.5	10.2	32	71
Hgb (g/L)	18.2	3.5	10.9	24.1
PCV (%)	52	11	22	65
TS (g/L)	8.8	1.1	5.8	10.7

*Values are rounded to the nearest tenth or whole number*.

In total, 100 individual crossmatches were performed with samples from 20 sea lions (8 animals from Playa Mann and 12 animals from Playa Los Marinos). The samples for the remaining 6 sea lions assessed were not utilized due to lack of sufficient sample or clotting of the sample. Each sea lion was crossmatched with between two and seven conspecifics depending on the randomization. Plasma samples utilized for crossmatching showed lipemia (*n* = 8) and hemolysis (*n* = 19). Of the 20 sea lions' auto-crossmatch test, 16 were negative for reaction (80%), one had positive microscopic agglutination (# 9–5%), two had hemolysis (# 6 and 19–10%), and one had both hemolysis and positive microscopic agglutination (# 7–5%). Of the paired crossmatches performed, 77% (77/100) were negative for any reaction, 12% (12/100) had positive microscopic agglutination, 4% (4/100) had hemolysis, 4% (4/100) had hemolysis and positive microscopic agglutination, and 3% (3/100) had a weak positive macroscopic agglutination reaction. Of the positive reactions observed, 5/12 of the positive microscopic agglutination, 3/4 of the hemolysis, 3/4 of the positive microscopic agglutination and hemolysis, and 3/3 of the weak positive macroscopic agglutination reactions involved at least one of the positive auto-crossmatch sea lions in the paired crossmatch. Detailed crossmatch results can be found in Table 3.

**Table 3:** Galápagos sea lion (*Zalophus wollebaeki*) crossmatching results of 100 combinations (50 major and minor paired matches) where the recipient donated plasma and the donor donated red blood cells for the major.

**Recipient sea lion**	**Donor sea lion**	**Major**	**Minor**	**Recipient sea lion**	**Donor sea lion**	**Major**	**Minor**
6	7	m+, hemolysis	Negative	5	6	Negative	m+, hemolysis
3	5	Negative	Negative	3	6	W+	Negative
9	10	Negative	Negative	5	12	Negative	Negative
11	12	Negative	Negative	15	16	Negative	Negative
3	9	Negative	Negative	17	18	Negative	Negative
5	7	Negative	m+	19	20	Negative	Negative
6	11	Hemolysis	m+	21	23	Negative	Negative
7	9	m+	m+	24	25	Negative	Negative
9	12	Negative	Negative	13	26	m+	Negative
10	11	Negative	Negative	15	18	Negative	Negative
3	7	Negative	Negative	19	24	Negative	Negative
5	11	Negative	Negative	16	21	Negative	Negative
6	9	Negative	Hemolysis	13	23	m+	m+
7	11	Negative	Negative	19	21	Negative	Negative
9	11	Negative	Negative	23	25	Negative	Negative
10	12	Negative	Negative	18	23	m+	m+
3	10	Negative	Negative	18	26	Negative	m+
5	10	Negative	Negative	16	17	Negative	Negative
6	12	m+, hemolysis	Negative	20	23	Negative	Negative
7	10	W+	Negative	17	23	Negative	Negative
3	11	m+, hemolysis	Negative	13	21	Hemolysis	Negative
5	9	Negative	Negative	18	19	Negative	W+
6	10	Hemolysis	m+	15	19	Negative	Negative
7	12	Negative	Negative	17	24	Negative	Negative
3	12	Negative	Negative	16	25	m+	Negative

*Components were reversed for the minor crossmatch. W+ and m+ are defined in Table 1*.

## Discussion

The physical examinations of the Galapagos sea lions evaluated in this study were largely unremarkable. Other than the ocular trematodes, no sea lions evaluated had outward signs of disease except one that was comparatively thin. On bloodwork, values for the sea lions were like those previously established in Galapagos sea lions (16). Both PCV and point of care HCT were measured during this study. The correlation in these values in Galapagos sea lions in unknown, however based off the values presented here, PCV trends lower than HCT. This suggests that similar to other wildlife species (17), point of care HCT may not be a reliable diagnostic tool in Galapagos sea lions. However, further analysis is needed. In the event a transfusion is performed in this species, the authors would recommend using PCV to monitor trends in the anemia following the transfusion.

The plasma samples utilized in this study had varying levels of hemolysis and lipemia. The lipemia in several samples was most likely due to a lack of fasting of animals prior to sample collection (18). It is unlikely that lipemic plasma would affect crossmatch results.

The hemolysis within the plasma samples is likely due to challenges during venipuncture. Challenges associated with venipuncture include resistance to restraint and anatomical vein location differences. However, type of tubes used, transport of the samples, time between collection of the samples and processing, and the centrifugation of the tubes can cause hemolysis and cannot be ruled out as causes of hemolysis in this study (19). *In vitro* hemolysis has been shown to increase the chances of a transfusion reaction in canine and feline medicine, thus utilizing blood products with <1% hemolysis is recommended (20). However, it is unknown how hemolysis in the plasma samples prior to crossmatch would affect the results. In this study, only 8% of crossmatches had evidence of hemolysis despite 95% of plasma samples having evidence of hemolysis prior to crossmatching. It could be argued that use of hemolyzed plasma would be inappropriate for a crossmatch test, however the difficulty of venipuncture makes hemolyzed samples very likely unless chemical restraint is utilized. This could be improved by calculating the percentage of hemolysis in each sample prior to crossmatching and selecting those that are <1%, as recommended for transfusions in small animals (20). It cannot be ruled out that hemolysis in the plasma caused spurious crossmatch results. If a transfusion is pursued in Galapagos sea lions, sedation may be warranted in the donor considering the challenges in venipuncture appreciated in this study. Sedation would limit venipuncture-related hemolysis and may allow use of other vessels (i.e*., jugular vein*) to possibly increase the volume of blood easily obtained.

Other wildlife species evaluated for crossmatch reactivity include green sea turtles and multiple shark species. Comparatively, intraspecies crossmatches for sharks were 100% negative and for green sea turtles there was ~50% negative reactions (11, 13). In cats, a retrospective study found ~85% of crossmatches to be negative (21). With the 77% negative reactions in Galapagos sea lions, it indicates that a transfusion reaction due to antibody-antigen binding is less likely, but other reactions are still possible. The positive microscopic agglutination (m+) and weak positive macroscopic agglutinations (W+) reactions suggest the likelihood of different blood types or natural alloantibodies in Galapagos sea lions; however, neither blood types nor natural alloantibodies have been previously studied in the Galapagos sea lion to the author's knowledge. The microscopic and macroscopic reactions could be due to previous exposure to conspecific blood due to intra-rookery fighting (22, 23). Therefore, it is uncertain whether evaluating adult males for crossmatch or transfusion potential would have different results due to an increased propensity for fighting.

Clinical use of crossmatching tests would be limited in animals with positive auto-crossmatch due to spurious results. Animals in this study with positive auto-crossmatches were over-represented in the reactive crossmatching pairs. Exclusion of these animals from the remainder of the study would likely change the results, however there were crossmatching pairs with one of the positive auto-crossmatch sea lions which produced a negative major and minor crossmatch. The cause of the reaction in pairs with one of the positive auto-crossmatch sea lions could be due to auto-agglutination, alloantibodies, or a spurious result. However, all W+ reactions contained a positive auto-crossmatch sea lion. This suggests that sea lions with a positive auto-crossmatch are at a higher risk of a transfusion reaction, although further understanding is warranted.

A consensus statement on transfusion medicine in canine and feline medicine discussed whether using a weak positive macroscopic reaction was more likely to cause a transfusion reaction than a completely compatible crossmatch (20). At the time of publication, there was not enough evidence to discern how a weak positive macroscopic reaction would affect the probability of a transfusion reaction and recommended using a completely compatible crossmatch when possible (20). Due to the lack of knowledge with regards to Galapagos sea lion blood transfusions, the recommendation would be to transfuse a completely compatible crossmatch. However, realistically detaining multiple wild animals for the length of time to determine a completely compatible crossmatch may prove to be challenging. In the event that only one animal is able to be selected as a donor, a crossmatch is still recommended. If the crossmatch returns as a weak positive, the authors recommend following the clinician's discretion as to whether or not move forward understanding that a weak positive macroscopic reaction increases the likelihood of a severe transfusion reaction.

If the crossmatch is completely compatible, the authors suspect the risk for a major transfusion reaction would be minimal, however this is yet to be studied. Regardless, if a Galapagos sea lion receives a blood transfusion from a conspecific, the animal must be constantly monitored for any signs of a transfusion reaction, regardless of crossmatch status. In companion animals, transfusion reactions have been recorded up to 12 days following a transfusion (24, 25). Monitoring a Galapagos sea lion for 12 days following a transfusion may present a logistical challenge if a pre-arranged space is not available. At this time, a transfusion may require significant pre-preparation.

At the time of this study, a blood transfusion has yet to be performed on a Galapagos sea lion to the authors knowledge. It is anticipated that a transfusion would be performed following anemia secondary to trauma or disease. Previously, parasitic infections (e.g., *hookworms)* have caused anemia in multiple species of pinnipeds while other parasitic species (e.g., *lungworms*) have caused coagulopathies in a species of pinniped (8). Although cases of anemia secondary to parasitism has been previously unreported in Galapagos sea lions, the species is susceptible to multiple species of parasites, including *Philophthalmus zalophi* which was found in the animals in this study. This ocular parasite has been previously evaluated for ocular pathology, clinical signs, and impact on the population (26, 27); however, its correlation to PCV has been left unstudied. Additionally, multiple other parasitic infections have been reported in Galapagos sea lions including *Mycoplasma* spp., *Parafilaroides* spp., and *Otostongylus* spp. (5, 28). Chronic infection with these parasites and yet to be discovered parasites may cause anemia in this species and thus warrant a transfusion as a component of the treatment.

## Conclusion

Crossmatch testing in juvenile Galapagos sea lions suggest a dearth of natural antibodies in the vascular system that would cause a transfusion reaction, so this option is viable in the event of a clinical need. A crossmatch prior to any transfusion is recommended out of caution, despite this, in emergency situations a transfusion could be pursued without a crossmatch in this species, since the risk of an immune-mediated one due to antigen-antibody binding is less likely. Finally, despite our small sample and the difficulty associated with obtaining samples, our findings are novel and important for heath studies on otariid species.

## Data Availability Statement

The original contributions presented in the study are included in the article/supplementary material, further inquiries can be directed to the corresponding author/s.

## Ethics Statement

The animal study was reviewed and approved by Universidad San Francisco de Quito (USFQ) under research permit PC-31-21.

## Author Contributions

TG, MP, GL, and DP-R contributed to conception and design of the study. TG and MP organized the database. TG and DP-R performed the statistical analysis. TG wrote the first draft of the manuscript. MP, GL, and DP-R wrote sections of the manuscript. TG, MP, DD, DA-R, JM-P, JT, GG, and GL worked in the field to collect samples. JT and DP-R approved necessary article work and field logistics. All authors contributed to manuscript revision, read, and approved the submitted version.

## Conflict of Interest

The authors declare that the research was conducted in the absence of any commercial or financial relationships that could be construed as a potential conflict of interest.

## Publisher's Note

All claims expressed in this article are solely those of the authors and do not necessarily represent those of their affiliated organizations, or those of the publisher, the editors and the reviewers. Any product that may be evaluated in this article, or claim that may be made by its manufacturer, is not guaranteed or endorsed by the publisher.
